# Beyond graphene: exploring the potential of MXene anodes for enhanced lithium–sulfur battery performance

**DOI:** 10.1039/d4ra02704c

**Published:** 2024-06-21

**Authors:** Zeshan Ali Sandhu, Kainat Imtiaz, Muhammad Asam Raza, Adnan Ashraf, Areej Tubassum, Sajawal Khan, Umme Farwa, Ali Haider Bhalli, Abdullah G. Al-Sehemi

**Affiliations:** a Department of Chemistry, Faculty of Science, University of Gujrat, Hafiz Hayat Campus Gujrat 50700 Pakistan asamgcu@yahoo.com; b Department of Chemistry, The University of Lahore Lahore Pakistan; c Department of Physics, Faculty of Science, University of Gujrat, Hafiz Hayat Campus Gujrat 50700 Pakistan; d Department of Chemistry, College of Science, King Khalid University Abha 61413 Saudi Arabia

## Abstract

The high theoretical energy density of Li–S batteries makes them a viable option for energy storage systems in the near future. Considering the challenges associated with sulfur's dielectric properties and the synthesis of soluble polysulfides during Li–S battery cycling, the exceptional ability of MXene materials to overcome these challenges has led to a recent surge in the usage of these materials as anodes in Li–S batteries. The methods for enhancing anode performance in Li–S batteries *via* the use of MXene interfaces are thoroughly investigated in this study. This study covers a wide range of techniques such as surface functionalization, heteroatom doping, and composite structure design for enhancing MXene interfaces. Examining challenges and potential downsides of MXene-based anodes offers a thorough overview of the current state of the field. This review encompasses recent findings and provides a thorough analysis of advantages and disadvantages of adding MXene interfaces to improve anode performance to assist researchers and practitioners working in this field. This review contributes significantly to ongoing efforts for the development of reliable and effective energy storage solutions for the future.

## Abstract 2

The high theoretical energy density of Li–S batteries makes them a viable option for energy storage systems in the near future.

## Graphical abstract

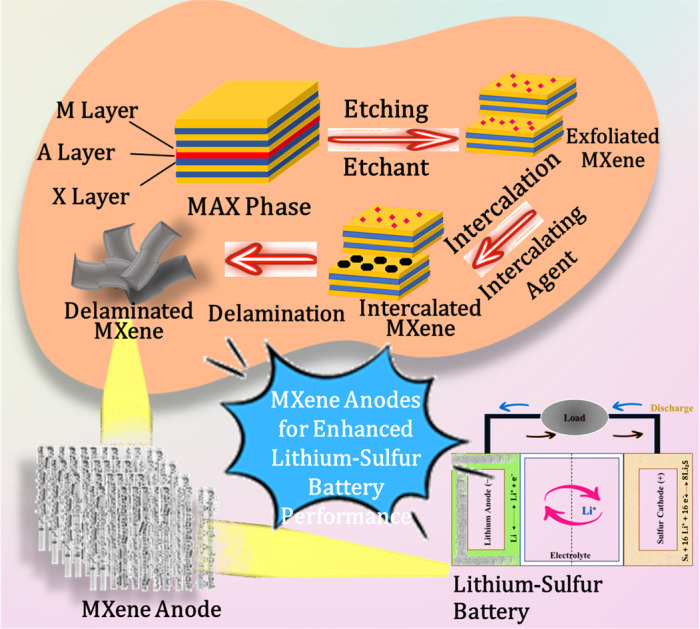

## 1. Introduction

MXenes are two-dimensional (2D) transition metal compounds that may transform lithium–sulfur (Li–S) batteries *via* carbides, nitrides, and carbonitrides.^1^ These innovative materials have attracted attention due to their unique structure and excellent electrochemical performance, which may help to address Li–S battery issues.^2^ Their practical implementation has been restricted by the limited electrical conductivity of sulphur and the breakdown of intermediate polysulfides caused by cycling. These 2D materials make good anodes because of their high electrical conductivity.^3^ The structured layers provide sufficient space for sulphur incorporation, thus effectively addressing the problem of polysulfide dissolution. MXenes are well known for their remarkable mechanical stability and strength, which prolong the life of Li–S batteries.^4^ The structural flexibility of MXenes allows for the accommodation of variations in Li–S battery volume during charge and discharge.^5^ Cycle stability and battery longevity are enhanced by this feature. As Li–S battery anodes, MXenes represent a significant advancement in energy storage.^6^ Because of its excellent electrical conductivity, sulphur retention, and structural stability, this method may increase energy storage by surpassing limitations of conventional Li–S batteries. With the development of battery technology, scientists may soon have workable and durable solutions.^7^

Lithium–sulfur batteries have recently been the focus of research due to their potential to revolutionise energy storage.^8–10^ Li–S batteries are anticipated to outperform and function better than Li-ion batteries due to their high energy density.^11,12^ The widespread use of Li–S batteries has been restricted by many challenges.^13^ Scientists are employing MXenes to address these problems and optimise the performance of Li–S batteries. Global concerns over the need for effective energy storage systems have been raised by the rapid growth of renewable energy sources, shift to electric cars, and need for dependable power storage solutions.^2,14,15^ Li–S batteries are a unique energy storage technology that might address several energy storage issues. Li–S batteries are more desirable because of several advantages.^13^ Consequently, they may have a greater energy density than lithium-ion batteries, enabling the development of more durable and long-lasting energy storage devices.^16^ Sulphur, an abundant and non-toxic cathode component, makes Li–S batteries more environmentally friendly. They are sustainable and aid the world's transition to greener energy.^17^ Sulphur outperforms lithium-ion batteries in terms of energy density. However, implementing the principle has been difficult.^18^ Sulfur's intrinsic insulating qualities prevent electrons from moving freely, which lowers battery performance. Sulphur may produce soluble polysulfides that can migrate away from the cathode as a result of the intricate electrochemical reactions that occur during cycling, which reduce capacity and shorten cycle life.^19^

**Fig. 1:**
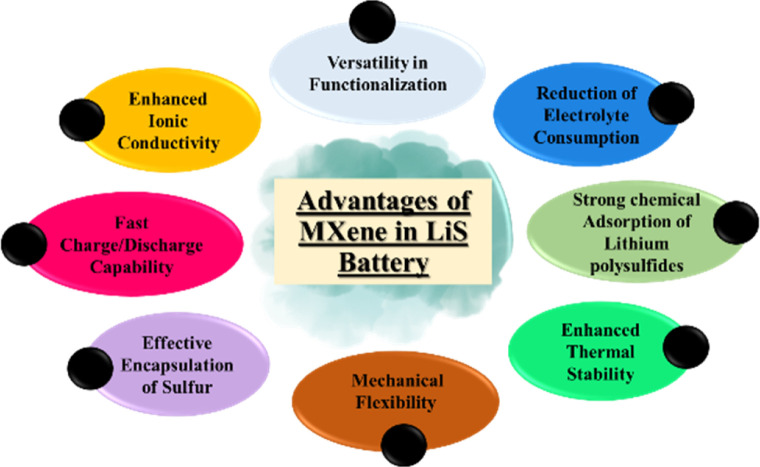
Imperative properties of MXene.

MXenes are a class of multilayer, ternary carbides and nitrides that were initially identified in 2011 by selectively etching a MAX phase. By eliminating the “A” layer, which is usually composed of aluminium, a two-dimensional structure was created.^20^ MXenes have excellent mechanical stability, adjustable surface chemistry, and electrical conductivity. These qualities stimulate the attention of researchers in the energy storage field and other fields.^21^ The strong conductivity of these materials overcomes the poor conductivity of sulphur, making them perfect anodes for Li–S batteries.^22^ MXenes improve battery electrochemical performance by accelerating electron transport while acting as anodes. According to the study, MXenes slow down the dissolution of polysulfide in Li–S batteries.^23^ By selectively interacting with sulphur species, MXenes may be surface modified to enhance polysulfide retention and trapping. The performance and efficiency of Li–S batteries are increased by this tailored surface chemistry.^24^ The various properties of MXene are shown in Fig. 1.

Despite volume changes, MXenes show exceptional structural stability when Li–S batteries are charged and drained. This property enables enhanced cycle stability and longer lifespans for Li–S batteries that use MXenes as the anode material.^25^ Understanding MXenes in Li–S batteries encourages environmental responsibility and sustainability. Li–S batteries are more affordable and have a higher energy density than lithium-ion batteries when it comes to renewable energy storage, electric vehicle power, and portable device usage.^26^ MXenes are revolutionizing energy storage, and additional research may improve efficiency and sustainability.^27^ For lithium-sulfur batteries, a stable lithium metal anode has polysulfide encapsulation: a layered MXene-protected lithium metal anode is an efficient polysulfide blocker.^28^ It has been reported that Ti_2_C-based MXenes are effective in enhancing the electrochemical performance of lithium–sulfur batteries. The introduction of the surface chemistry characteristics of MXenes enhances the performance, whereas concerns related to the favourable functionalized surface during the charge and discharge process exist.^29^ A simple approach for creating 3D S-CNT@MXene cages is further proposed to address challenges in the LiS batteries. Three-dimensional cages with conductive networks in contact with each other can increase sulfur active site accessibility, reduce electrode resistance, and promote reaction rates.^30^ Tailoring MXene (2D-Ti_3_C_2_)-derived TiN with well-defined facets yields an efficient bidirectional electro catalyst for high-performance Li–S batteries.^26^ Chemical etching is employed to obtain the delaminated Mo_2_CT_*x*_ MXene nano sheets that are utilized as sulfur hosts. The amplified shuttle effect and comparatively slow recharge ability of polysulfides are major drawbacks in the practical implementation of lithium–sulfur batteries.^31^ An MXene/MoS_2_/SnS@C flower structure used as the functional intercalation of Li–S batteries was designed to enhance the synergistic electrocatalytic processes involved in sulfur conversion. The MXene framework forms a three-dimensional conductive backbone that constrains the morphology of the polysulfides and promotes charge transfer.^32^ A sample of multi-hetero-structured MXene/NiS_2_/Co_3_S_4_ with rich S-vacancies was fabricated using a hydrothermal and high-temperature annealing process. The MXene sheet not only serves as a mechanical barrier but also enhances the conductivity and adsorption capability of the catalyst NiS_2_/Co_3_S_4_ double active centre, accelerating the conversion of LiPSs.^33^

This article reviews the latest research on MXene anodes in lithium–sulfur batteries. This study aims to explain the unique features of MXenes that make them suitable anode materials. The features include mechanical stability, chemical compatibility with lithium–sulfur systems, and electrical conductivity. This study critically analyses the volume growth, cycle stability, and potential unfavorable reactions of MXenes that might influence the performance of lithium–sulfur batteries. This article summarizes MXene-based anodes in lithium–sulfur battery systems and discusses future approaches and applications.

## 2. Lithium–sulfur battery technology

**Fig. 2:**
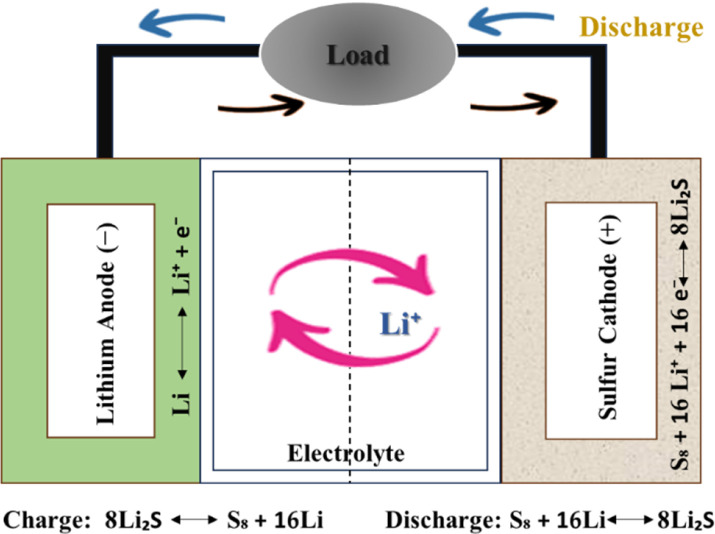
Basic assembly of lithium sulfur battery.

Although cheaper than lithium-ion batteries, lithium–sulfur (Li–S) batteries have gained popularity as energy storage alternatives due to their high theoretical energy density. If you want more energy and less pollution, Li–S batteries are chosen.^28^ Sulphur, unlike lithium cobalt oxide, is a potential lithium-ion cathode. Removing heavy metals from cathodes reduces health risks, battery manufacturing and disposal environmental effects. Research and development are focused on solving Li–S battery issues and improving their capabilities.^34^ These innovative batteries combine sulphur and MXenes to store energy efficiently and sustainably *via* electrochemical reactions.^35^ Lithium and sulphur react during a Li–S battery's discharge cycle to produce electricity.^36^ Lithium anodes, generally Li metal or Li-ion intercalation materials, initiate the process, while sulphur functions as the cathode. Li^+^ ions go from the anode to the cathode *via* the electrolyte, and this process is reversed during charging. The motion of lithium ions generates battery output.^37^ At the cathode, intermediate lithium polysulfides (Li_2_S_*x*_), where *x* is the number of sulphur atoms in the polysulfide, are created when lithium ions react with sulphur (S8).^38^ This implies that Li-ion batteries hold energy by lowering Li-ions through a reversible process. During this process, electrons are liberated, producing an electrical current with various uses.^39^ The electrons produced by sulphur reduction processes may provide an electrical current that can power electronics or operate machines by connecting them to an external circuit.^40^ The battery's capacity to produce electricity is based on this electron flux. The basic assembly of the Li–S battery is shown in Fig. 2.

One must reverse the electrochemical events that occur during discharge to recharge a Li–S battery. The charging process's basic operation involves many phases.^41^ When lithium polysulfides (Li_2_S_*x*_) receive external electrical energy, they undergo oxidation reactions. Lithium ions are released into the electrolyte, and sulphur molecules (S8) are formed as a consequence of this process.^42^ The lithium anode's electrolyte concurrently transports lithium ions from the cathode to the anode. These lithium ions are absorbed by the lithium metal or lithium-ion intercalation anode.^43^ Electrons from the external circuit enter the anode during charging and mix with lithium ions. This procedure returns the lithium ions to their initial condition for the next discharge cycle.^44^ The anode material in Li–S batteries is made of MXene compounds. In the process of charging, electrons from the external circuit enter the anode and mix with lithium ions. In addition to improving the battery's performance during the next discharge cycle, this procedure fixes typical problems with Li–S batteries by restoring the lithium ions to their initial conditions.^45^ Sulphur's weak electrical conductivity is a concern with Li–S batteries, whereas the great conductivity of MXenes addresses this issue. Additionally, MXene materials efficiently immobilise Li_2_S_*x*_ produced during discharge and charge operations.^46^ Even with volume differences from cycling, MXenes are mechanically stable. Due to their mechanical resilience, Li–S batteries have better cycling stability and longevity.^47^ Li–S batteries with MXene anodes work because of the synergy between lithium ions, sulphur cathodes, and the particular characteristics of MXenes. MXenes improve the performance and solve problems in typical Li–S battery technology.^48^ Research and development in this sector are constantly changing the operating principles and practical uses of these cutting-edge energy storage technologies.^49^

## 3. MXene material properties and synthesis

### 3.1 Overview of MXene materials

**Fig. 3:**
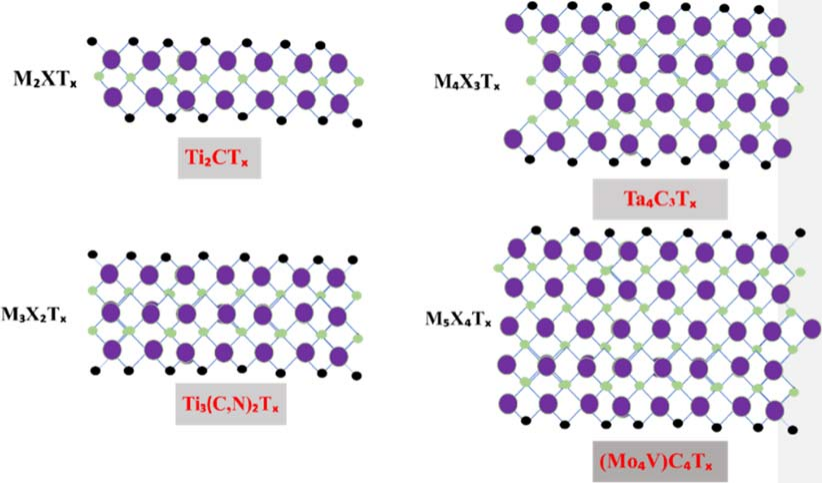
Two-dimensional structures of MXene with different transition metals.

Professor Yury Gogotsi and Professor Michel W. Barsoum discovered MXene, a new 2D material, in 2011 with their colleagues at Drexel University.^50^ The basic formula of MXene can be expressed as M_*n*+1_X_*n*_T_*z*_ or M1.33XT_*z*_ (*z* = 1, 2, 3), where TX shows terminal functional groups, including oxygen, hydroxide, and fluorine; M represents early transition metals, such as Sc and Ti; and X represents carbon or nitrogen elements.^51^ Generally, MAX-phase substances and their correspondents are used to produce MXene.^52^ Max phases are layered ternary carbides and nitrides^53^ with a general formula (M_*n*+1_AX_*n*_),^54^ where ‘M’ represents the transition metal, ‘A’ represents the group IV–V element, and ‘X’ represents either carbon or nitrogen.^55^ The MXene family did not demonstrate its existence before 2011 but has significantly grown in prominence from the chemistry and application viewpoint,^56^ and scientific reporting affiliated with MXene nearly doubles each year.^57^ In recent years, numerous two-dimensional substances have been fabricated, including phosphorene, silicon germanane, hexagonal boron nitride, and transition metal dichalcogenides.^58^ MXene composed of different metals is shown in Fig. 3.

The majority of already known 2D materials until 2011 were poor or not conductors of electricity and exhibited low carrier concentrations.^59^ MXene materials have numerous fascinating physico-chemical properties,^59^ such as excellent electrical conductivity (4.52 × 10^−4^ S m^−1^);^60^ a tunable band gap;^61^ large negative zeta potential;^62^ good flexibility;^63^ and chemical,^64^ constructional,^65^ optical and magnetic properties, along abundant active catalytic sites.^66^ MXene materials also have good mechanical properties, and their hardness and strength moderately improve by increasing ‘n’.^67^ The most abundantly studied MXene is Ti_3_C_2_T_*x*_, which exhibits superb versatility, processing ability, and design ability due to its unique physical and chemical properties.^68^ MXenes possess numerous features, such as high surface area, hydrophilicity and a lesser diffusion barrier, due to the presence of a large number of functional groups.^69^ The electronic conductivity of MXene materials is affected by the position of metals and functionalities, such as O, F, and OH, present on the surface of MXenes.^70^

### 3.2 Synthesis techniques

**Fig. 4:**
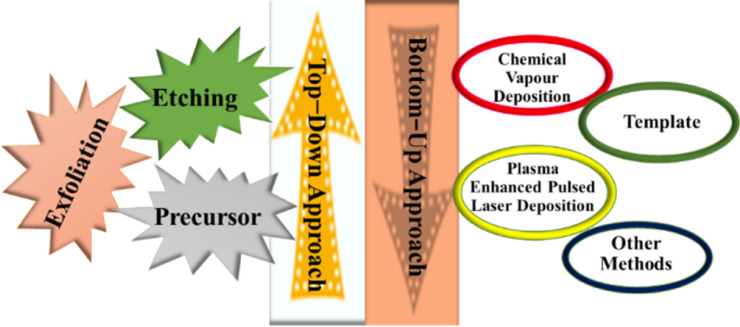
Different methods for the synthesis of MXene.

In general, MXenes can be fabricated using either bottom-up or top-down methods.^65^ The prevailing procedure in top-down involves acid etching, whereas chemical vapor deposition is the key approach in the bottom-up method,^71^ which can manufacture good-quality films on substrate.^72^ Electro catalytic properties were affected when different methodologies modified the structure or surface termination of MXene materials.^73^ The top-down approach is the famous synthetic method, starting from the Max phase.^65^ MXene materials are acquired from their precursor MAX phases by chemical exfoliation.^51^ In the beginning, by mixing the elemental powders of M, A, and X in specific atomic ratios at high temperatures, the formation of an MAX phase and sample densification occurs after hot or cold pressing.^74^ After the fabrication of the MAX phase, the initial step involves the etching of the 3D Max phase using a potent etchant, typically hydrofluoric acid. M–A bonds are weaker than M–X bonds, allowing for the selective etching of M–A bonds.^65^ The various methods for the synthesis of the MXene are presented in Fig. 4.

**Fig. 5:**
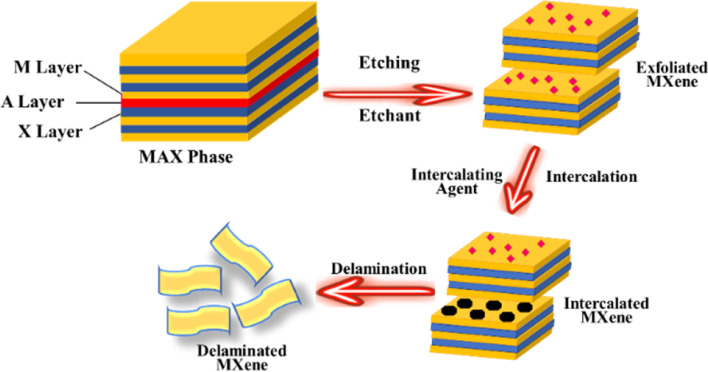
Synthesis of MXene by employing the acid exfoliation method (top-down approach).

Etchants are necessary to disrupt the robust chemical bonds that exist between elements A and M in the Max phase.^74^ Several techniques utilized for the synthesis of MXene have been reported, namely HF etching, *in situ* etching, molten fluoride etching, non-fluorine etching, and electrochemical etching.^75^ In 2021, Zamhuri and colleagues pioneered the synthesis of the initial MXene by immersing Ti_3_AlC_2_ powder in 50% concentrated hydrofluoric acid (HF) for 2 hours at room temperature. This process led to the comprehensive dissociation of the Max phase.^65^ Three lattice structures of MXenes, consisting of 3, 5 and 7 atomic layers, represented as M_2_X, M_3_X_2_ and M_4_X_3_, respectively, can be derived from the Max phase.^70^ Confirmation of the conversion from the MAX phase to MXene can be achieved through X-ray diffraction (XRD) and energy-dispersion spectroscopy (EDS).^74^ In the fabrication of MXene, the method of etching with HF is presently employed, but it is essential to emphasize that larger concentrations of HF can be dangerous^75^ as corrosive agents, making their handling and disposal harmful.^76^ Verger *et al.* (2019) explored the feasibility of using a mixture of hydrochloric acid (HCl) and lithium fluoride (LiF) for etching Max phase materials. This approach generates small amounts of *in situ* HF.^77^ Ammonium hydrogen bifluoride (NH_4_HF_2_) and ammonium fluoride are commonly employed in the synthesis of Ti_3_C_2_ from Ti_3_AlC_2_.^78^ An alternative technique involves combining Ti_4_AlN_3_ powder with a specific mixture of molten fluoride salts, such as NaF, KF, and LiF. For half an hour, the mixture is blended at 550 °C.^65^ A recent work by Li *et al.* (2021) presented a method for creating a Zn-based MAX phase with a chlorine-terminated, fluorine-free surface. MXene accomplished this by reacting the Lewis acidic molten salt with the MAX phase *via* a replacement reaction mechanism.^79^ Electrochemical exfoliation has been extensively used for 2D materials, such as graphene and phosphorene. In a recent study, Chaturvedi *et al.* (2023) showed how to synthesise Ti_2_CT_*x*_ in a three-electrode cell by electrochemical etching.^75^ The acid exfoliation method for MXene synthesis is depicted in Fig. 5.

**Fig. 6:**
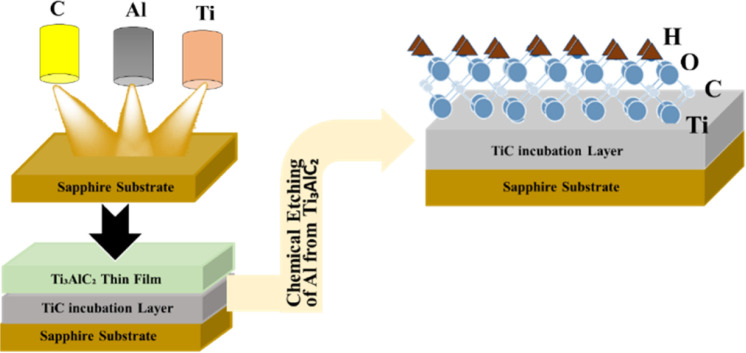
Synthesis of MXene using the chemical deposition method (bottom-up approach).

It is noteworthy that specific types of MXene, such as MoC and MoN, cannot be synthesised using the top-down approach.^80^ Achieving precise control over particle lateral size, defects, and tribological properties is possible through bottom-up approaches, where atoms and molecules are assembled to form complex 2D MXene structures.^81^ Chemical vapour deposition (CVD) is a commonly employed method for MXene synthesis, providing several benefits over traditional top-down approaches.^78^ Plasma enhancement is utilised in the traditional chemical vapour deposition process to improve material quality and enable synthesis at reduced temperatures. The synthesis methods for MXenes are continuously evolving due to the rapid expansion of the domain.^78^ MXene synthesis *via* a bottom-up approach is shown in Fig. 6.

### 3.3 Surface functionalization

Functionalized Ti_3_C_2_T_2_ (T = N, O, F, S, and Cl) demonstrated the same metallic conductivity as bare Ti_3_C_2_. Among all the Ti_3_C_2_T_2_ units, Ti_3_C_2_S_2_, Ti_3_C_2_O_2_, and Ti_3_C_2_N_2_ have moderate adsorption power, which hinders lithium dissolution and shuttling. This Ti_3_C_2_T_2_ showed excellent electro catalytic activity for Li_2_S decomposition. Li_2_S decomposition barrier noticeably decreased from 3.390 eV to ∼0.4 eV using Ti_3_C_2_S_2_ and Ti_3_C_2_O_2_*via* fast Li^+^ diffusivity.^25^ A boost in the performance of lithium–sulfur batteries is achieved using Ti_3_C_2_T_*x*_, where T for the surface termination is –O, –F, and –OH as MXene nanosheet coating, which is a commercial Celgard membrane. Contrarily, the Ti_3_C_2_T_*x*_ MXene has an ultrathin two-dimensional structure and can form a uniform coating layer with a minimum mass loading of 0.1 mg cm^−2^ and a thickness of a mere 522 nm. At the expense of the enhanced electric conductivity and the efficient encapsulation of polysulfides, the lithium–sulfur battery.^82^ Simultaneously, the Li–S battery suffers from lithium polysulfide (LiPS) shuttling influence and a slow kinetics reaction. In this way, nano hybrid N-doped MXene-CoS_2_ (N-MX-CoS_2_) is developed through an *in situ* sulfidation strategy. The MXene-CoS_2_ chaperoned N-doped separator presents an excellent initial specific capacity of 1031 mA h g^−1^ at 1 °C, high-rate performance and outstanding cycle stability (0.52% per cycle).^83^ The simple formation of the thiourea-actuated wrinkled nitrogen and sulfur co-doped functionalized MXene (NSMX) in the separator to enhance ion diffusion and conversion kinetics is now feasible for high-energy LSBs. It should be mentioned that the LSBs with NSMX-modified separators showed a high specific capacity of 1249 mA h g^−1^. The LSBs delivered an excellent reversible capacity of 600 mA h g^−1^.^84^ 3D S-CNT@MXene cages lower the electrode resistance and speed up the reaction rate. Consequently, the 3D S-CNT@MXene cage electrode emerges with a superior discharge capacity of 1375.1 mA h g^−1^. The first benefit lies in its high-rate capacity (910.3 and 557.3 mA h g^−1^) and outstanding heat transfer stability. Impressively, the composite electrode displays around zero capacity fading (656.3 mA h g^−1^), indicating the highest cycling stability reported so far among the whole Li–S cells.^30^ The MXene-based materials along with their different properties are presented in Table 1.

**Table 1:** MXene-based material and its properties

Material type	Material synthesis	Material efficiency	Optimization of Li–S batteries	References
Ti_3_C_2_T_*x*_ MXene	Ti_3_AlC_2_ treated with LiF/HCl, air-dried, stirred in DMSO, centrifuged, rinsed, sonicated, and dispersed in water	Multilayered 2D Ti_3_C_2_T_*x*_ nano sheets used in Ti_3_C_2_T_*x*_-PP separators with mass loadings 0.16–0.016 mg cm^2^	Maintained 640 mA h g^−1^ capacity after 200 cycles at 1 °C with 0.079% decay per cycle	85
3D porous Ti_3_C_2_T_*x*_ MXene/rGO (MX/G) hybrid aerogel	GO from Hummers' method, Ti_3_C_2_T_*x*_ synthesized *via* LiF/HCl etching, hydrothermal method, and freeze-drying	Sulfur content ∼45 wt%, higher sulfur loading evaluated	High capacity of 1270 mA h g^−1^ at 0.1 °C, extended cycling life up to 500 cycles with 0.07% decay per cycle, and high areal capacity of 5.27 mA h cm^−2^	86
Ti_3_C_2_ nanosheet/glass fiber composite	Few-layered Ti_3_C_2_*via* ultrasonication and vacuum filtration for composite	Extremely thin, single-layered or few-layered Ti_3_C_2_ nanosheet on GF	Initial discharge capacity of 820 mA h g^−1^ at 0.5 A g^−1^ and 721 mA h g^−1^ after 100 cycles	87
Ti_3_C_2_-lithium film anode	Atomic layers of Ti_3_C_2_ prepared by LiF in 6 M HCl, ultrasonication, freeze-drying, rolling and folding for anode	Low overpotential, well-confined lithium plating	Overpotential of 32 mV at 1.0 mA cm^−2^, 1.5% increase in 200 cycles, and flat voltage profiles	88
MXene debris-coated eggshell membrane (MXene/ESM)	Ti_3_C_2_ MXene hydrothermally reacted, ESM treated with HCl, coated with MXene debris	Enhanced cycling stability compared to polypropylene separator	Discharge capacity of 1321 mA h g^−1^ at 0.1 °C, and capacity retention of 74% after 250 cycles at 0.5 °C	89
3D MnO_2_ nanosheets@delaminated-Ti_3_C_2_ (MNSs@d-Ti_3_C_2_) aerogel	d-Ti_3_C_2_*via* etching, ultrasonic treatment, MnO_2_ on d-Ti_3_C_2_	High specific surface area, mesoporous structure, and robust conductive pathway	Initial discharge capacity of 1140 mA h g^−1^, 615 mA h g^−1^ at 2.0 °C, and 0.06% decay per cycle over 500 cycles at 1.0 °C	90
Functionalized Ti_3_C_2_T_2_ MXenes (T = N, O, F, S, Cl)	Total energy calculations for T-saturated Ti_3_C_2_ monolayers	Reduced Li_2_S decomposition barrier and fast Li^+^ diffusivity	Significantly decreased decomposition barrier and efficient Li^+^ transport	25
Nanodot-interspersed Ti_3_C_2_T_*x*_ nanosheet (TCD-TCS)	Ti_3_AlC_2_ treated with HF, dispersed in hydrosol, autoclaved, and freeze-dried	High surface polar sites and enhanced structural integrity	Discharge capacity at medium sulfur loading, the high volumetric capacity of 1957 mA h cm^−3^, and the high areal capacity of 13.7 mA h cm^−2^	91
Heterostructures of layered covalent triazine framework on Ti_3_C_2_ MXene nanosheets (CTF/TNS)	Ti_3_C_2_ nanosheets *via* ultrasonic exfoliation, monomer heated, mixture ground, and washed with HCl	High sulfur loading and efficient electron/ion transport	Reversible capacity of 1441 mA h g^−1^, 0.014% decay rate over 1000 cycles, and 94% capacity retention after 100 cycles	92
CO_2_-oxidized Ti_3_C_2_T_*x*_ MXenes components	Ti_3_C_2_T_*x*_ prepared by LiF/HCl, CO_2_ oxidation at 900 °C	High coulombic efficiency and retention of capacity	Capacity of about 900 mA h g^−1^ after 300 cycles at 1 °C	93
GO-d-Ti_3_C_2_T_*x*_ MXene aerogels with 3D reticular structure	Ti_3_C_2_T_*x*_*via* etching, centrifugation, freeze-drying, sonication, mixed with GO, and autoclaved	Rapid LiPSs capture, improved capacity and cycling performance	Discharge capacity of 1039 mA h g^−1^, 0.048% decay rate per cycle, and the areal capacity of 4.3 mA h cm^−2^	94

## 4. MXene as anode material

MXene, a recently discovered category of 2D materials, has garnered considerable interest in the energy storage sector due to its exceptional characteristics and wide range of uses.^95^ According to density functional theory (DFT) studies, V_2_CT_*x*_ MXene has promising features for use in energy storage applications, such as lithium-ion batteries and supercapacitors.^96^ When Na ions are intercalated or adsorbed onto the surface of MXene nano sheets, the interlayer spacing between the layers of MXene increases from 7.1 to 10.1 Å.^97^ This V_3_C_2_/graphene heterostructure showcases an increased capacity and fast charge/discharge rates, indicating its promise as a highly effective electrode for ion batteries. This shows great potential, especially in the field of sodium-ion batteries.^98^ MXenes are recognised for their electrochemical activity, enabling them to exhibit pseudo-capacitance, resulting in a higher capacity than electrodes in double-layer capacitors and faster kinetics than intercalation-type electrodes.^99^ Garg and colleagues (2020) were among the first to publish the potential applications of MXenes as anodes in lithium-ion batteries. This newly discovered material demonstrates a surface area expansion that is about ten times larger than graphene.^97^ These applications have been utilised to enhance the performance and stability of separators, electrolytes, and electrodes.^100^ Several methods have been proposed to develop stable, dendrite-free metal anodes using MXene. The host designs are inspired by MXene, while the substrates are designed to be metalphilic. MXene-modified metal surfaces, MXene array construction, and MXene-decorated separators or electrolytes are all addressed.^101^ MXene has been extensively studied because it plays a significant role in various emerging composites. This composite anode effectively decreases the resistance to lithium ion movement at the interface between the lithium metal anode and the garnet solid-state electrolyte.^102^ MXene's surface functionalities allow it to undergo multiple reactions while maintaining its electrical conductivity.^103^ When O-terminated MXenes react with Mg, Ca, or Al, they decompose into bare MXenes, offering high capacities and excellent rate capabilities. The bare MXenes also demonstrate an outstanding performance.^104^ MXene has quickly gained recognition as the “next wonder material” following its introduction, setting it apart from other two-dimensional materials in the field.^105^

### 4.1 MXene anode in lithium–sulfur batteries

**Fig. 7:**
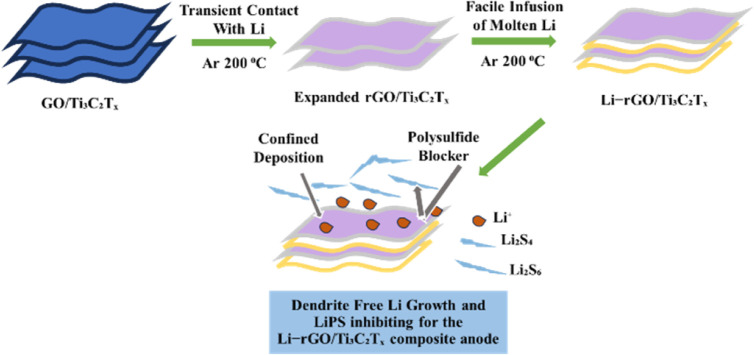
Representation of lithium metal anode with layered MXene, an effective polysulfide blocker for lithium–sulfur batteries.

Lithium–sulfur batteries (LSBs) have become a focal point in the energy storage industry due to their remarkably high theoretical energy density and the cost efficiency of their active materials,^106^ which depend on the sulfur-lithium reversible redox reactions,^34^ and represent a promising alternative power source compared to existing lithium-ion batteries.^107^ Due to the natural availability of sulfur, high theoretical specific capacity (1675 mA h g^−1^), and high energy density (2670 W h kg^−1^), the lithium–sulfur battery is regarded as one of the most promising electrical energy storage devices.^18^ The significant volume change in S/Li_2_S (80%), which may damage the electrode structure, and the lithium dendrites on the anode developing throughout the charge/discharge process might cause the battery to short circuit.^2^ The limited utilization of active materials and unstable cycling performance are the issues that lithium–sulfur batteries are currently facing.^108^ There is a further decrease in performance due to the infamous shuttle effect. Porous-structured carbon materials are used as S hosts. Good electrical conductivity is provided by the carbon framework, and the shuttle effect is reduced by the porous structure.^109^ Carbon-based materials can only offer modest confinement towards lithium polysulfides, resulting in capacity degradation and low rate capability that occur when lithium sulphur batteries increasingly diffuse into the electrolyte.^110^Fig. 7 illustrates the lithium metal anode used as a polysulfide blocker for lithium–sulfur batteries.

To address the shuttle issue, Zhao and his colleagues (2020) investigated the lithium nitrate (LiNO_3_) addition, and electrolyte formulae such as ionic liquid and localized high-concentration electrolytes are also suggested.^40^ Experimental findings reveal that the Li-rich Li–Mg alloy is a promising anode material for Li–S batteries, as it forms a strong passivation layer on its surface, reducing side reactions.^111^ The electrochemical characteristics of Li–Mg alloys generated by kinetically controlled vapour deposition or direct alloying show that the tendency for Li dendrite development is greatly decreased on an electrode made of Li–Mg alloy.^112^ The development of macroscopic pores can be effectively reduced by the alloying process. However, the alloy also exhibits a restriction in delithiation due to diffusion control.^113^ MXenes are now among the most promising choices for anode materials.^114^ MXenes can intercalate massive ions and offer extraordinary capacities at high power rates over hundreds of cycles after building composites with graphene, metal oxides, transition metal dichalcogenides, and silicon.^103^ In a super capacitor, the working electrode created from free standing MXene paper exhibits a remarkable capacitance of approximately ≈490 F g^−1^ at 1 A g^−1^, a value that stands as one of the highest reported for super capacitor electrodes based on MXene.^115^ The cathodes, anodes, and separators of Li–S batteries use MXene-based materials, which have demonstrated the theoretical and experimental significance of high surface polarity and rich surface chemistry in poly-sulfide trapping. They are superior in reducing poly-sulfide shuttling and increasing sulphur utilization.^3^ Due to their unique properties, MXenes, a new category of 2D transition metal carbides and nitrides, have gained attention as promising materials for lithium–sulfur battery anodes. Their performance analysis requires a sophisticated approach that uses many graphs.^116^ The following graphs are crucial to comprehending how MXenes impact Li–S battery performance. The cyclic voltammetry graphs depict the relationship between the current and applied voltage throughout a voltage sweep. The electrochemical reactions occurring at the interface between an electrode and an electrolyte can be greatly understood from these curves. When anodes composed of MXene material have cathodic peaks on their CV curve, this usually means that the sulfur species has been converted to lithium sulfide (S_8_ → Li_2_S_2_) and has decreased, while the solid electrolyte interface layer, or SEI layer, has formed. Anodic peaks demonstrate how sulfur is created during the charging of the battery by oxidizing Li_2_S_2_.^117^ A region surrounded by the cyclic loop of CV indicates the electrode's potential specific capacity. The CV plots of pristine and cycled MXene electrodes make it easy to observe variations in the peak locations and peak currents. These differences suggest changes regarding the reversibility along with the kinetics of the process.^118^

### 4.2 Electrochemical performance

**Fig. 8:**
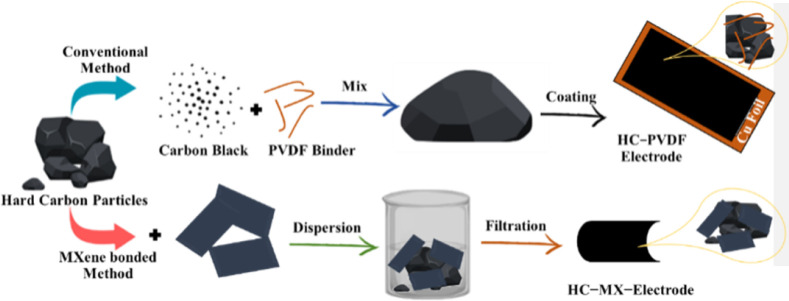
MXene-bonded hard carbon film as an electrode material.

It is generally established that there is a strong association between material size and electrochemical performance. Smaller sizes result in higher electrochemically active regions and shorter ion diffusion distances. The connection stated greatly improves electrochemical performance.^119^ MXene and MXene-based nanomaterials show significant promise for energy storage applications.^120^ This two-dimensional MXene nano sheet serves as an outstanding conductive additive that improves electrochemical stability.^121^ MXenes, mainly Ti_3_C_2_T_*x*_ and its derivatives, are particularly effective in improving the electrochemical performances of lithium–sulfur (Li–S) batteries and are potential candidates for next-generation energy in various storage systems. MXenes have an optimal structure for electron conductivity and polysulfide adhesion, which are two of the most critical issues in Li–S battery science.^122^ A new 3D architectural electrode was created by integrating MXene and carbon nanotubes (CNTs) to overcome the issues of restacking and decreasing the specific capacity observed in MXene as an anode material.^123^ The utilization of lighter electrode materials frequently leads to an increase in capacity,^124^ as shown in Fig. 8.

MXenes are used in double-layer and redox-type ion storage, ion transfer control, electrodeposition substrates, batteries, and super capacitors. They increase electrode and electrolyte separator stability and performance.^100^ The synthesis of over 30 MXenes with controlled layer spacing and superconductivity expanded their use in electrode materials.^125^ MXene-based electrode materials have increased specific capacity and rate capability; they reduce or even completely stop the growth of dendrites on the metal anodes, extending the life of rechargeable batteries.^126^ One-component MXene electrodes struggle to attain high specific capacity, efficient ion/electron transport, and stable compatibility in electrochemical environments. Introducing nanomaterials between MXene layers increases electrochemical performance.^127^ Recent studies have shown that the creation of heterostructure nanocomposite from 2D MXenes and transition metal sulfides (TMS) or transition bimetal sulfides can enhance the specific capacitance, long-term cycling stability, and rate capability of MXene-based electrode materials.^62^ Compared to pristine TiNb_2_O_7_, the TNO@MXene composite has significantly improved lithium storage characteristics, including high reversible capacity (346.4 mA h g^−1^ at 0.1 °C), cycling stability (92.3% capacity retention after 500 cycles at 10 °C), and superior rate capability.^128^ The electrochemical efficacy of the Fe–Ti_3_C_2_T_*x*_ electrode is significantly improved (564.9 mA h g^−1^ at 50 mA g^−1^ at −10 °C). Over 500 cycles, the cycling stability of Fe–Ti_3_C_2_T_*x*_ was determined to be 418.8 mA h g^−1^ at 200 mA g^−1^ at −10 °C.^129^

With a high specific capacitance of 583 F g^−1^ at 1 A g^−1^, a decent rate capability of 82.5%, and an outstanding cycle stability of 96.5% at 5 A g^−1^ for over 5000 cycles, the heterogeneous 2D-layered MoS_2_/MXene nanohybrid MMX electrode displays a hybrid-type capacitance behavior.^130^ Fe–Ti_3_C_2_T_*x*_ electrode exhibits greatly enhanced electrochemical performance (564.9 mA h g^−1^ at 50 mA g^−1^ under −10 °C), surpassing that of pristine Fe–Ti_3_C_2_T_*x*_ (77 mA h g^−1^). The cycling stability of Fe–Ti_3_C_2_T_*x*_ for over 500 cycles (418.8 mA h g^−1^ at 200 mA g^−1^ under −10 °C). This work is expected to provide a guideline for developing brand-new MXene-based electrode materials with a high capacity for energy storage.^129^ Perpendicular MXene–Li arrays with tunable MXene walls and constants showed promise for dendritic-free and high-capacity lithium metal batteries due to their high specific capacity (2056 mA h g^−1^), long cycle life (1700 h), good rate capabilities of up to 2500 cycles (at 20 mA cm^−2^), and deep stripping and plating capability of up to 20 mA h cm^−2^.^131^ The unique two-dimensional sulfur-decorated Ti_3_C_2_ MXenes, as well as the self-enhanced kinetic and hybrid energy storage processes, are responsible for their superior electrochemical performance.^132^ The electrochemical properties in terms of current density, reversible capacity and cycle number are presented in Table 2.

**Table 2:** Electrochemical characteristics of MXene as anode for lithium battery

Material	Current density	Reversible capacity (mA h g^−1^)	Cycle number (mA g^−1^)	Reference
Ti_2_C	1	110	80	133
Ti_2_C	1	123.6	75	134
Ti_3_C_2_ MXene	—	84	200	135
Ti_3_C_2_	200	203	500	136
Ti_3_C_2_	100	47.9	3000	137
Porous Ti_3_C_2_	3500	220	1000	138
Ti_2_C_2_T_*x*_	30	100	50	139
Ti_2_CNT_*x*_	310	500	1000	140
V_2_C	500	243	500	141
V_4_C_3_	1000	125	300	142

## 5. Enhanced battery performance

**Fig. 9:**
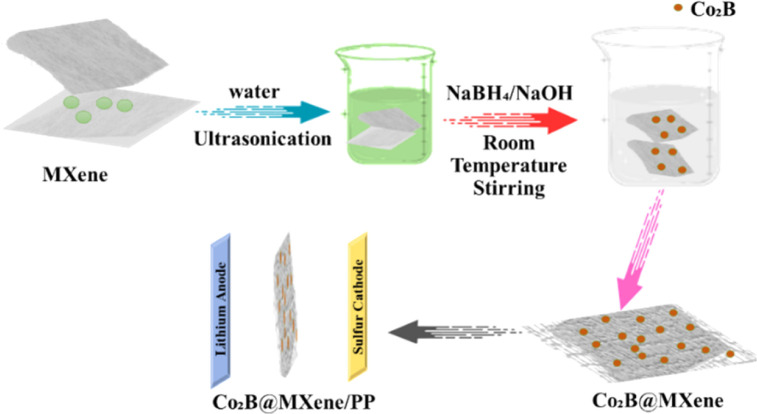
Interfacial electrical interaction of cobalt boride@MXene for high-performance lithium–sulfur batteries.

Energy storage plays a crucial role in shaping our future amidst rapid technological progress. Lithium–sulfur batteries are a promising option for high-energy-density storage systems positioned to revolutionize the electric vehicle and renewable energy sectors.^30^ Developing suitable anode materials has proven to be a challenging endeavour in the progression of Li–S battery technology.^143^ MXene, a recent innovation, may increase Li–S battery performance.^144^ High energy density, cost-effectiveness, and environmental friendliness make lithium–sulfur batteries an attractive energy storage option. However, several challenges have hindered their widespread adoption.^145^ Low sulfur-based cathode coulombic efficiency and cycle stability are important challenges.^146^ Li–S systems are not compatible with conventional graphite anodes for Li-ion batteries. MXene is used in energy storage because of its special qualities. These materials are perfect Li–S battery anodes because of their electrical conductivity, mechanical strength, and chemical stability.^147^ Battery performance significantly improves when MXene is used as the anode.^148^ The cobalt boride@MXene's interfacial electrical interaction for high-performance lithium–sulfur batteries is illustrated in Fig. 9.

**Fig. 10:**
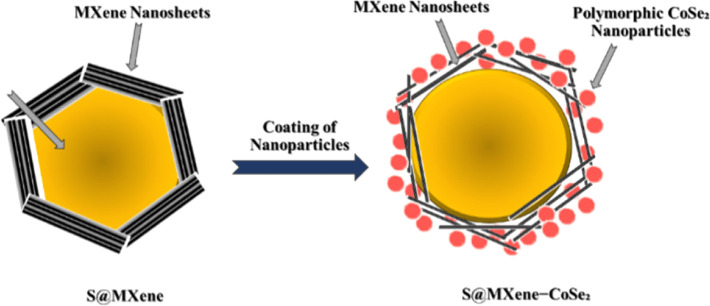
Representation of CoSe_2_-decorated MXenes as cathodes in lithium–sulfur batteries.

Overall, Li–S battery performance is enhanced by MXene's higher electrical conductivity.^149^ MXene permits high current densities for rapid charging and discharging, minimising energy loss and boosting battery power density in contrast to graphite anodes.^92^ Because of their longer lifespan and quicker charging times, Li–S batteries are gradually outperforming Li-ion batteries.^150^ The mechanical properties of MXene also enhance the structural stability of Li–S batteries. During cycling, sulphur cathodes expand and contract, resulting in electrode deformation and failure.^151^ These issues are resolved by MXene's robust mechanical properties, which maintain the anode's structure for the course of the battery's life.^152^ MXene anodes are recognised for their chemical stability, along with these benefits. When in contact with the reactive elements of the battery, they show reduced susceptibility to degradation.^153^ In addition, the widespread availability of MXene materials in nature, and their straightforward synthesis methods position them as a cost-effective option for anode materials.^154^ With the increasing demand for energy storage solutions, the cost-effectiveness of advanced battery technologies, such as Li–S, is becoming crucial for their widespread adoption.^155^ The cost advantage of MXene can lower Li–S battery prices overall, increasing its competitiveness in the market.^156^ In conclusion, MXene has proven to be a groundbreaking anode material for lithium–sulfur batteries. All these factors synergistically enhance battery performance: strong electrical conductivity, polysulfide-trapping ability, mechanical strength, and chemical stability.^157^ Despite a few remaining challenges, such as MXene's compatibility with different sulfur-based cathode materials and the necessity for large-scale production methods, the encouraging findings from research laboratories suggest that MXene is crucial for the progress of Li–S battery technology.^158^ Anticipate remarkable advancements in lithium–sulfur battery performance as researchers refine and optimise the technology, moving us closer to a sustainable and efficient energy storage solution for the future.^159^Fig. 10 depicts the CoSe_2_-decorated MXenes as the cathode in lithium–sulfur batteries. Current and future batteries require anode materials with good conductivity and capacity. Batteries with sulfur (S) active ingredients in the electrode have an excellent specific capacity of around 1675 mA h g^−1^ (theoretical). The deposition or dissolution of sulfides (Li_2_S_*m*_, *m* = 1, 2, 4, 6, 8) in electrolytes can significantly impact the cycle property and specific density of LSB. To address these problems, numerous efforts have gone into developing anode protection techniques, researching the latest developments, and discovering effective electrode additives.^160^ Different chemicals, especially carbon-based chemicals, have larger surface areas. These chemicals can be utilized in the electrochemical and lithium sulfide processes, and they have been considered very promising additions. Other strategies, such as doping of N or B, are needed to improve the competences of carbon materials in lithium sulfur batteries.^85^ In the meantime, the creation of novel additives may improve LSB performance. Several materials have been available recently and are utilized in the anodes of LSB, including metal oxides (MOs), transition metal–organic frameworks (MOFs), and transition metal dichalcogenides (TMDCs). In our growing mobile world, this can significantly improve ease of use and user experience. MXene-based lithium-ion (Li–S) batteries have a high capacity and the potential to be economically viable, which makes them perfect for large-scale systems that store energy.^161^ Furthermore, it has been shown that metalization materials can enhance Li–S battery performance.^22^

### 5.1 Mechanistic insights

**Fig. 11:**
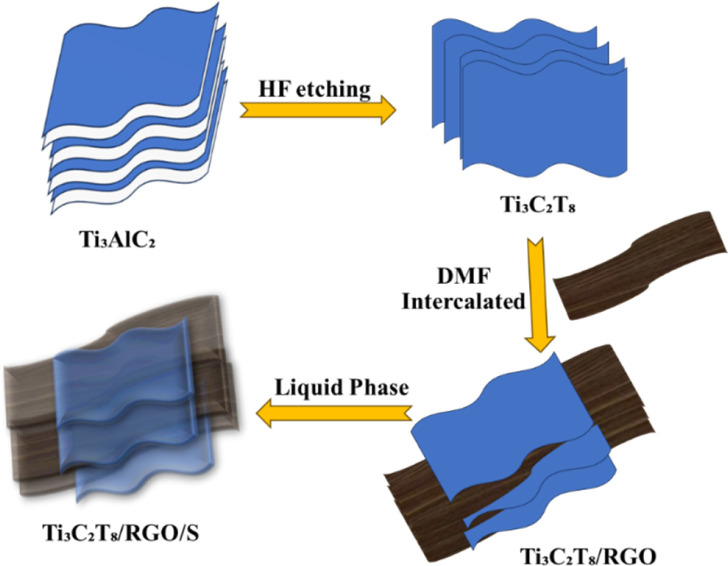
Depiction of MXene/reduced graphene for trapping sulfur and polysulfides.

Lithium–sulfur batteries are considered a potential solution to meet the increasing demand for high-energy-density options in the quest for more effective and environmentally friendly energy storage systems.^162^ The use of MXene materials has revolutionised this industry. These compounds have improved Li–S battery performance and revealed their mechanisms.^163^ MXene materials have unique qualities and structures that make them ideal for improving Li–S battery efficiency.^164^ Finding the mechanics underlying MXene materials in Li–S batteries has revealed electrochemical reactions and has led to better, more lasting energy storage technologies.^165^ Trapping and immobilising polysulfides (Fig. 11) in Li–S batteries are difficult; therefore, this mechanistic finding is significant. Sulfur-based cathodes create soluble lithium polysulfides that migrate to the anode during cycling, reducing capacity and performance.^82^ Due to their two-dimensional structure and immense surface area, MXene materials prevent polysulfide diffusion.^166^ Spectroscopy and microscopy showed that MXene is a polysulfide “sponge”, demonstrating the process. MXene traps polysulfides to improve Li–S battery performance and endurance.^166^

MXene materials provide excellent electrical conductivity, which is important for Li–S batteries. High current densities for quick charging and discharging require conductivity higher than that of graphite.^167^ Energy dissipation and battery power density decrease due to this constraint. By facilitating quick charge transfer, MXene materials boost Li–S battery energy production and make them ideal for many applications.^168^ MXene's remarkable mechanical capabilities are crucial for Li–S battery structural integrity. Sulphur cathodes expand and compress during cycling, deforming and perhaps failing. MXene's superior mechanical strength solves these difficulties.^169^ Through scanning electron microscopy, MXene-supported anodes have been shown to preserve their structural integrity throughout the battery's lifetime. Understanding how MXene materials retain anode structure is essential for improving Li–S battery performance.^170^

The chemical stability of MXene materials in Li–S batteries provides significant mechanistic insights. When in touch with the battery's reactive components, MXene resists breakdown better than other anodes.^171^ Improved chemical stability extends the calendar life, which is important for electric cars and renewable energy storage. Through extensive investigation using multiple analytical techniques, MXene's chemical stability has been shown to affect Li–S battery efficiency.^122^ The use of MXene materials in Li–S batteries has improved their performance and opened the road for energy storage technological developments.^172^ Researchers are improving MXene material synthesis, compatibility with other cathode materials, and large-scale manufacturing techniques to commercialize this approach.^173^ MXene illuminates Li–S battery issues, such as polysulfide migration, poor conductivity, and structural instability.^174^ These findings have impacted Li–S battery research and development, predicting future advancements. Their energy storage competitiveness and sustainability should improve with this advancement. MXene compounds might improve Li–S batteries and accelerate energy transition.^174^

### 5.2 Environmental considerations

Lithium–sulfur batteries have received a lot of attention lately because of their potential to completely change the energy storage industry. There are many environmental and sustainable benefits associated with using MXenes as the anode material in these batteries.^174^ MXenes have shown a lot of potential for increasing Li–S battery lifespan and efficiency.^89^ Lithium and sulphur work together to provide a larger potential energy density because Li–S batteries have a higher energy density than regular Li-ion batteries.^175^ Li–S batteries store more energy in smaller areas. MXenes can boost Li–S battery energy density as anodes. Energy storage systems using Li–S batteries reduce their environmental impact. Sulphur, a key ingredient of Li–S batteries, is cheap and abundant, making it an eco-friendly option.^176^ Li–S batteries lack harmful heavy elements, such as cobalt, unlike lithium-ion batteries. This reduces environmental and ethical issues related to battery mining and production.^177^ The environmental impact of Li–S batteries may be reduced with MXenes, which are made of easily accessible components.^178^ Long-term environmental consequences, durability, and recycling should be considered when assessing batteries. Li–S batteries may last longer than lithium-ion batteries. MXenes improve Li–S battery stability as an anode.^179^ MXenes may help manage sulphur expansion and contraction, extending battery life, decreasing replacements, and preserving waste.^175^ Recycling and reuse are essential to sustainability. Due to its lower usage of hazardous and valuable components, Li–S batteries are easier to recycle than lithium-ion batteries.^180^ MXenes may improve recycling. These 2D materials may be reused to make batteries due to their stability. This reduces environmental effects by avoiding fresh raw material extraction and processing, thereby contributing to a circular economy.^181^ Safety is essential to sustainability. Due to the decreased thermal runaway danger, Li–S batteries are safer than lithium-ion batteries.^182^ The thermal stability and flame-retardant qualities of MXenes reduce battery fires and explosions, thereby improving energy storage system safety and sustainability.^183^

## 6. MXene stability in Li–S batteries

Stability is crucial to the lifetime and performance of lithium–sulfur batteries using MXene anodes.^184^ MXene may solve Li–S battery difficulties. However, stability across many charge–discharge cycles remains the major goal.^4^ Li–S batteries using MXene as the anode dissolve lithium polysulfides (LiPS), causing stability concerns. LiPSs are formed during battery usage and may migrate into the electrolyte, reducing their cycle life and capacity.^185^ MXene may mitigate this issue. During sulfur-to-lithium sulphide conversion, LiPS production at the anode is decreased by its good electrical conductivity. The large surface area of MXenes collects and immobilises LiPS, preventing them from escaping into the electrolyte.^186^ MXene has benefits but does not solve LiPS migration and disintegration. A few LiPS may still enter the electrolyte, threatening battery stability.^187^ Thus, research is developing new electrolyte formulations and separator materials to reduce LiPS migration and improve stability.^188^ The stability and structural integrity of the MXene anode during charge–discharge cycles are concerns. Li–S reactions cause volume variations in the anode material, which may deteriorate or pulverise it. Due to its endurance and flexibility, MXene may assist in solving these problems.^189^ Its two-dimensional structure allows it to handle volume fluctuations better than three-dimensional anodes. In rare circumstances, cycle expansion and contraction may destroy structures. Composite materials and new electrode designs are being investigated to improve MXene anode mechanical stability.^190^ Thermal stability is also important, and Li–S batteries may overheat, causing thermal runaway, which is a serious failure.^191^ High thermal conductivity makes MXene efficient in heat dissipation. Cell designs, safety features, and sophisticated thermal management systems must be improved to provide Li–S battery thermal stability.^192^ Long-term storage stability is also significant. MXene-anode Li–S batteries may self-discharge and lose capacity after complicated storage. The problem is LiPS reactivity, which absorbs lithium ions even when the battery is off.^193^ Increasing Li–S battery stability and reducing self-discharge are persistent problems. MXene is a promising Li–S battery anode.^194^ For MXene to reach its full potential in Li–S batteries and ensure long-term stability and reliability, collaboration among materials scientists, chemists, and engineers is essential. Overcoming stability issues is crucial for the successful implementation of Li–S batteries with MXene anodes in various applications, such as energy storage and electric vehicles.^195^ It is crucial to thoroughly investigate the challenges related to MXene stability during cycling in lithium–sulfur batteries when used as an anode material for the advancement of this promising energy storage technology.^196^ MXenes possess several benefits, such as robust electrical conductivity and the ability to facilitate sulphur growth. However, there are also notable challenges that need to be addressed. MXenes demonstrate the ability to undergo structural and chemical modifications during repeated charge and discharge cycles, leading to enhanced electrochemical stability. Instability can lead to a loss of electrical conductivity and structural integrity.^197^ Researchers are currently working on enhancing the electrochemical stability of MXenes through modifications to their surface chemistry and structure.^198^ Understanding the interaction between sulphur and MXenes is essential for optimizing the performance of Li–S batteries. Sulphur reacts with MXenes during cycling, forming undesirable intermediate compounds.^199^ Lowering the battery's reversible capacity may affect cycle stability. Controlling and optimising sulfur-MXene interactions is difficult.^31^ MXene nanoparticles may agglomerate, lowering the lithium-ion adsorption surface area and active sites. This may reduce battery capacity and performance. Researchers are using advanced materials engineering to reduce particle aggregation.^200^ Due to their sensitivity, MXenes in Li–S batteries require careful electrolyte and solvent selection. Certain electrolyte and solvent combinations may damage the MXene anode material, reducing stability and performance. This challenge requires finding acceptable electrolyte systems.^201^ For practical use, Li–S batteries must function consistently. MXene stability issues connected to long-term performance require an anode material that can withstand many charge–discharge cycles without deteriorating.^202^ Researchers are using surface functionalization, hybrid nanocomposite design, and improved manufacturing methods to address these challenges.^203^ Electric cars, renewable energy storage, and portable electronics might benefit from the high energy density and sustainability of Li–S batteries.^204^

## 7. Strategies for MXene-based anode improvement

Li–S batteries are inexpensive and have a high theoretical energy density, making them a good energy storage option. Significant difficulties exist, especially with anode materials.^205^ Li–S battery anodes using MXenes seem promising. The high electrical conductivity, large surface area, and mechanical resilience of MXenes make them excellent for overcoming traditional anodes.^129^ Researchers have investigated ways to improve MXene-based Li–S battery anodes to maximize their potential.^206^ Optimal Li–S battery anode performance requires the right sulphur incorporation and MXene compatibility.^207^ Sulphur species may be linked to MXene surface functional groups. Recent investigations have found ways to link sulphur and MXenes for stable electrochemical reactions. Surface modifications and functionalization generate chemical linkages that prevent sulphur species diffusion during charge/discharge cycles.^208^ Developing nanostructured composites through the combination of MXenes with other nanomaterials, such as carbon nanotubes, graphene, or metal oxides, provides a synergistic approach.^209^ These composites exhibit superior mechanical stability, electrical conductivity, and lithium-ion diffusion. They help manage the volume changes that happen during lithium-ion intercalation, minimizing structural damage and enhancing battery longevity.^210^ Dealing with the production of soluble polysulfides during discharge remains a significant hurdle in Li–S batteries. Polysulfides may migrate from the cathode to the anode, leading to a decrease in capacity over time.^211^ To solve this problem, scientists have developed surface coatings for MXene-based anodes. The coatings effectively capture and immobilize polysulfides, preventing their migration and reducing the impact of the “polysulfide shuttle” effect. Materials such as polymers or metal oxides, which have a strong attraction to polysulfides, are commonly utilized in these coatings.^212^ Enhancing the porosity of MXene-based anodes leads to a significant improvement in their performance. High surface area and porosity facilitate increased sulphur loading, improved electrolyte infiltration, and faster ion diffusion.^213^ Researchers have used various methods to incorporate porous patterns into MXene-based anodes, such as templating, chemical etching, and controlled oxidation. The enhancement of electrochemical activity and battery performance is evident in these structures.^214^ Choosing the right electrolyte is crucial for the performance of Li–S batteries and significantly affects the behavior of the anode. Scientists have explored tailored electrolyte formulations to enhance their compatibility with MXene-based anodes.^215^ Electrolytes possessing suitable ionic conductivity, stability, and chemical composition play a crucial role in enhancing anode electrochemical performance while mitigating undesired side reactions.^216^ There is a growing emphasis on environmental and sustainability considerations in the advancement of Li–S batteries. MXenes are known to be recyclable materials that house valuable transition metals.^217^ Investigations are currently underway to explore strategies for sustainable MXene production and recycling to minimize the waste and environmental impact of Li–S battery manufacture.^218^ A crucial phase in the development of MXene-based anodes is converting laboratory research into practical commercial uses.^219^ Assessing the practicality and reliability of these anodes involves real-world testing, such as pilot-scale production and battery testing. MXenes may be viable Li–S battery anodes due to their unique characteristics. MXenes are improving energy storage technologies in collaboration with current research and development.^220^ Researchers are making progress towards high-performance, cost-effective, and sustainable Li–S batteries. These batteries will be vital to greener, more efficient energy storage. MXene-based anodes may improve Li–S anodes and boost energy storage technology.^221^

## 8. Future directions in advancing lithium–sulfur batteries

Lithium–sulfur batteries provide an abundance of intriguing opportunities and technical difficulties that require further research and development. The significance of developing robust cathode composites made of carbon and investigating hybrid electrolyte methods to progress the creation of long-lasting Li–S batteries. The value of using electrocatalysts and novel electrode materials lies in solving the problems associated with lithium polysulfides. However, Li–S batteries are far more promising compared with these little advancements. This joint endeavour might revolutionize high-capacity, sustainable energy storage technologies by exploring material genomes and integrating machine-learning methods. Significant progress is expected in the development of Li–S batteries using materials based on MXene, in line with these overall objectives. Tackling technical issues includes increasing sulphur loading as well as utilizing and enhancing the stability of the materials for lithium anodes. The development of MXene-based Li–S batteries has accelerated owing to high-throughput material screening and optimization that use computational design and material genome techniques. All things considered, Li–S batteries seem to have a bright future, particularly when the surface functionalization of MXene materials is prioritized. This combination of advanced surface modification methods and the ongoing development of MXene-based composite anodes may lead to promising advancements in energy storage technology. The next mission emphasizes how important it is to continue materials science and engineering research to overcome current obstacles and achieve MXene's full potential in Li–S batteries.

## 9. Conclusions

This study highlights the significant progress achieved in enhancing the performance of MXene-based anodes for this specific energy storage application. Enhancing and developing MXene interfaces have become essential approaches to solving problems in lithium–sulfur batteries. This study has provided insights into the potential advantages and drawbacks of these novel materials through a detailed analysis of the vast range of MXene modifications and their impact on anode performance. Customized MXene interface designs have been shown to be successful in reducing issues such as electrode instability, polysulfide shuttling, and volume expansion, which eventually improve battery performance. It is now possible to enhance the anode's electrochemical properties by including MXene derivatives, functional additives, and nanocomposites. This leads to a longer cycle life, better cycling stability, and increased utilization of sulphur. Moreover, an improved understanding of MXene's interactions with lithium and sulphur species has made it possible to create strategic interface designs that enhance the control of the charge/discharge process. As with any new technology, there are always challenges. The long-term stability, affordability, and scalability of MXene-based anodes require further investigation. To accelerate the commercialization of MXene interfaces in lithium–sulfur batteries, these problems need to be resolved in future research. In summary, the comprehensive analysis of the MXene interface design provides guidance for further research aimed at optimizing the potential of these materials for the advancement of lithium–sulfur battery technology. The study underscores MXene's potential as the leading option for customized anode creation, hence enhancing the lifespan and performance of lithium–sulfur batteries.

## Data availability

Data will be provided on request.

## Author contributions

Zeshan Ali Sandhu and Muhammad Asam Raza have supervised the project, Kainat Imtiaz and Adnan Ashraf have collected the data, Areej Tabasum and Sajawal Khan have compile the data, Ali Haider Bhalli has drafted the final version, Umme Farwa has drawn the whole figures while Abdullah G. Al-Sehemi have helped in polishing the final draft and give the financial assistant.

## Conflicts of interest

Authors declare that they have no conflict or competing interest.
